# The Morphological Anatomy of the Menisci of the Knee Joint in Human Fetuses

**DOI:** 10.4274/balkanmedj.2016.0081

**Published:** 2017-12-01

**Authors:** Esra Koyuncu, Gülnur Özgüner, Kenan Öztürk, Cemil Bilkay, Ahmet Dursun, Osman Sulak

**Affiliations:** 1 Department of Anatomy, Süleyman Demirel University School of Medicine, Isparta, Turkey

**Keywords:** Foetus, meniscus, Placenta, fetal development

## Abstract

**Background:**

Development of the foetal period of the meniscus has been reported in different studies.

**Aims:**

Evaluation of lateral and medial meniscus development, typing and the relationship of the tibia during the foetal period.

**Study Design:**

Anatomical dissection.

**Methods:**

We evaluated 210 knee menisci obtained from 105 human foetuses ranging in age from 9 to 40 weeks’ gestation. Foetuses were divided into four groups, and the intra-articular structure was exposed. We subsequently acquired images (Samsung WB 100 26X Optical Zoom Wide, Beijing, China) of the intra-articular structures with the aid of a millimetric ruler. The images were digitized for morphometric analyses and analysed by using Netcad 5.1 Software (Ak Mühendislik, Ankara, Turkey).

**Results:**

The lateral and medial meniscal areas as well as the lateral and the medial articular surface areas of the tibia increased throughout gestation. We found that the medial articular surface areas were larger than the lateral articular surface areas, and the difference was statistically significant. The ratios of the mean lateral and medial meniscal areas to the lateral and medial articular surface areas, respectively, of the tibia decreased gradually from the first trimester to full term. The most common shape of the medial meniscus was crescentic (50%), and that of the lateral meniscus was C-shaped (61%).

**Conclusion:**

This study reveals the development of morphological changes and morphometric measurements of the menisci.

The menisci are two cartilaginous structures that deepen the proximal articular surface of the tibia (1,2,3). These fibrous cartilage structures reduce the stress on the tibia and contribute to the congruence of the knee joint during stance and gait (1,2,3,4,5). Meniscus injuries and meniscal tears are more prevalent among active children who participate in sports, and their frequency increases during adolescence (3,6). The snapping knee syndrome is frequently observed in children and is related to meniscal area. Congenital anomalies of the menisci are rare. The most common developmental anomaly is discoid meniscus (4,7). A number of researchers have reported a relation between meniscal injuries and discoid meniscus (4,8,9). During the foetal period, discoid meniscus is more commonly observed in the lateral meniscus (LM) (9). Different theories have been proposed about the formation of discoid meniscus. Some researchers mentioned that discoid meniscus occurs due to failure of central resorption during the foetal period (4,9), and others noted that no discoid meniscus occurred in the foetal period (7,8).

In adults, the morphology of the tibia differs from that of the femur; therefore, the morphologies of the lateral and medial menisci (MM) are also expected to differ (4). Structural incompatibility between the femur and tibia may be the reason for many clinical problems, particularly MM injury. In adults, the shape, size and thickness of the LM are different to those of the MM (9,10). At the same time, it has been reported that the LM of the tibia in the adult surface footprint of the MM is larger than the area covered by the tibial surface. We examined the medial and lateral femoral condyles in foetal development and found that that the height and width of the lateral femoral condyle were greater than those of the medial condyle (11). These results are similar to those of studies carried out in adults (11). Therefore, it is expected that the medial and lateral menisci also differ during the foetal period (11).

Secondary to the increase in the use of CT and MRI in research, there has been a surge in studies on the anatomy of and variations in the menisci in adults (9). However, there are very few studies on the normal anatomy and anomalies of the menisci in new-borns (3,7,8). In such studies the foetal menisci were classified (3,8), the areas of the foetal menisci were measured or foetal and adult meniscal areas were compared (4). Therefore, in the present study the foetal menisci and tibia were examined morphometrically in a large series. In addition, the anatomic dissection method provides more reliable and accurate results than imaging methods applied in the foetal period.

To clarify the probable developmental origin of the aetiology, investigation and quantitative examination of the normal development of knee joint morphology in the prenatal periods are essential; however, such studies related to human foetuses are limited. In the current study, we examined the morphological changes in the meniscus and the tibial plateau quantitatively in human foetuses. The aim of the study was to examine the areas of the menisci, the superior lateral and medial articular surface areas of the tibia and the ratios of the menisci to the corresponding areas of the articular surfaces of the tibia. We analysed the distances between the anterior and posterior horns of the lateral and MM, the distance between the anterior and posterior horns of each meniscus and the shapes of the menisci during the foetal period.

## MATERIALS AND METHODS

This study was carried out on 210 knees of 105 human foetuses (52 males and 53 females), aged between 9 and 40 weeks of gestation, obtained with consent from the families from Maternity and Children’s Hospital between 1996 and 2011. Only foetuses without any external pathology or anomaly were used. The data collection procedure was approved by the Ethics Board of the Faculty of Medicine of the University. The post-mortem dissection procedures were ethically approved by the Turkish Ministry of Health and thus were in accordance with statutory regulations.

Gestational ages of the foetuses were determined by using crown rump length, bi-parietal width, head circumference, femur length and foot length (12). Foetuses were assigned to one of four groups according to their gestational ages: Group 1 (first trimester), Group 2 (second trimester), Group 3 (third trimester) and Group 4 (full term) were comprised of foetuses aged 9-12 weeks, 13-25 weeks, 26-37 weeks and 38-40 weeks, respectively.

**FIG. 1.**
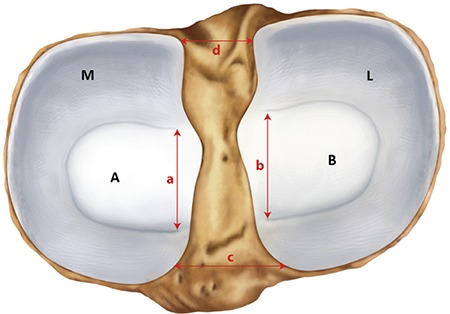
Site of measurement of the menisci and the superior articular surface areas of the tibia. M: area of the medial menisci; L: area of the lateral meniscus; A: medial articular surface areas of the tibia; B: lateral articular surface areas of the tibia; a: distances between the anterior and posterior horns of the medial menisci; b: distances between the anterior and posterior horns of the lateral menisci; c: distances between the anterior horns of the menisci; d: distances between the posterior horns of the menisci

Initially, the knee region was anatomically dissected to expose the intra-articular contents. This was followed by image acquisition (Samsung WB 100 26X Optical Zoom Wide, Beijing, China) of the intra-articular structures with the aid of a millimetric ruler. The images were digitized for morphometric analyses and analysed by Netcad 5.1 Software (Ak Mühendislik, Ankara, Turkey). Areas of the lateral and MM and the superior lateral and medial articular surface areas of the tibia were measured (Figure 1). The ratio of the lateral meniscal area to the lateral articular surface area of the tibia and that of the medial meniscal area to the medial articular surface areas of the tibia were calculated. The distances between the anterior and posterior horns of the lateral and the MM as well as between the anterior horns of the menisci and between the posterior horns of the menisci were measured (Figure 1).

**FIG. 2.**
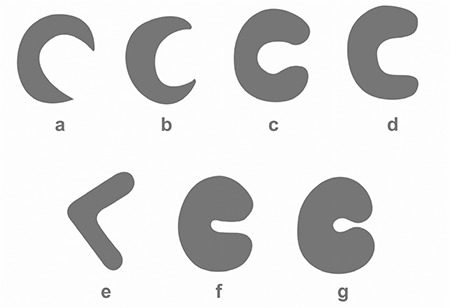
Diagram showing the shape of the lateral and medial menisci. a: crescentic (semilunar) type; b: sickle-shaped type; c: C-shaped; d: U-shaped; e: V-shaped; f: incomplete discoid type; g: complete discoid type

We morphologically classified the lateral and MM based on previous classifications in the literature (3,8,9). The shapes of the menisci were determined as crescentic (semilunar), sickle shaped, C shaped, U shaped, V shaped, incomplete discoid or complete discoid as follows (Figure 2) (3,8,9). In the crescentic (semilunar) type, the anterior and posterior horns and the body of the meniscus are thin, and the gap between the horns is wide. In the sickle-shaped type, the anterior and the posterior horns are thin, the body of the meniscus is wide and the gap between the horns is wide.

In the C-shaped type, the widths of the horns and body are similar, and the tips of the horns are rounded and close to each other. In the U-shaped type, the widths of the horns and body are similar, the tips of the horns are rounded and the gap between the horns is wide. The V-shaped type has a shape resembling the letter V. The incomplete discoid type has a deficiency at the centre and between the horns of the menisci. The complete discoid type has a defect at the centre of the meniscus and no gap between the horns.

SPSS (17.0) statistical package was used to compute the arithmetic means and standard deviations of the parameters (SPSS Inc, Chicago, IL, USA). The level of significance was set at α=0.05. Parametric variables were expressed as mean ± standard deviation. A Student’s t-test was used to compare the parametric variables between sexes and sides (all cases combined). One-way ANOVA and Bonferroni’s post-test were used for comparisons between groups. For nonparametric data, a chi-square (χ^2^) test was used to compare percent distributions among groups, and P and χ^2^ values are presented in the relevant tables of the Results section.

## RESULTS

Mean LM areas were 6.44 mm², 17.52 mm², 42.71 mm² and 59.36 mm² in the first, second and third trimester and full-term groups, respectively. Mean MM areas were 5.79 mm², 17.04 mm², 40.44 mm² and 60.38 mm² in the first, second and third trimester and full-term groups, respectively. Mean lateral and medial meniscal areas increased throughout gestation, and there were significant differences between groups (p<0.05, Table 1).

Mean lateral articular surface areas of the tibia were 7.90 mm², 22.41 mm², 54.07 mm² and 78.76 mm² in the first, second and third trimester and full-term groups, respectively. Mean medial articular surface areas of the tibia were 9.19 mm², 27.32 mm², 65.43 mm² and 101.76 mm² in the first, second and third trimester and full-term groups, respectively. There was a significant increase in the areas of the lateral and medial articular surfaces of the tibia throughout gestation (p<0.05, Table 1). When the lateral and medial articular surface areas of the tibia were compared, we found that the medial articular surface areas were larger than the lateral articular surface areas, and the difference was statistically significant (p>0.05, Table 1).

The ratio of the mean lateral meniscal area to the lateral articular surface areas of the tibia was computed. The ratio was 0.81 in the first trimester, 0.78 in the second trimester, 0.78 in the third trimester and 0.75 at full term. The ratio of the mean medial meniscal area to the medial articular surface areas of the tibia was 0.63 in the first trimester, 0.62 in the second trimester, 0.61 in the third trimester and 0.59 at full term (Table 2).

The distance between the anterior and posterior horns of the menisci was measured. The distance between the anterior and the posterior horns of the LM increased from 0.81 mm in first trimester to 1.84 mm in the second trimester, 2.60 mm in the third trimester and 3.60 mm at full term. The distance between the anterior and posterior horns of the MM was increased from 1.92 mm in first trimester to 3.55 mm in the second trimester, 5.57 mm in the third trimester and 7.23 mm at full term (Table 3). Results showed that the distance between the anterior and posterior horns of the menisci was increased throughout gestation, and the increases were statistically significant. Further, the distance between the anterior and posterior horns was greater for the MM, and the difference was found to be statistically significant (p<0.05).

We also measured the distance between the anterior horns of the menisci as well as the distance between the posterior horns. The mean distance between the anterior horns was 1.97 mm in the first trimester, 3.71 mm in the second trimester, 5.97 mm in the third trimester and 8.02 mm at full term (Table 3). The mean distance between the posterior horns was 1.34 mm in the first trimester, 2.15 mm in the second trimester, 3.30 mm in the third trimester and 4.40 mm at full term (Table 3). Both distances increased throughout gestation, and these increases were statistically significant (p<0.05). The distance between the posterior horns was lower than the distance between the anterior horns, indicating that the posterior horns are closer to each other.

**FIG. 3.**
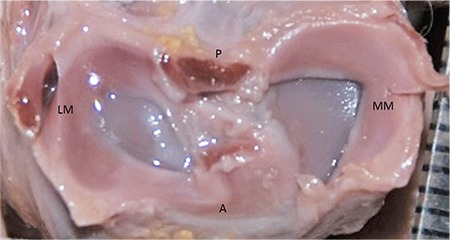
30-week-old foetus, the LM crescentic shaped, the MM V shaped. LM: lateral meniscus; MM: medial menisci

**FIG. 4.**
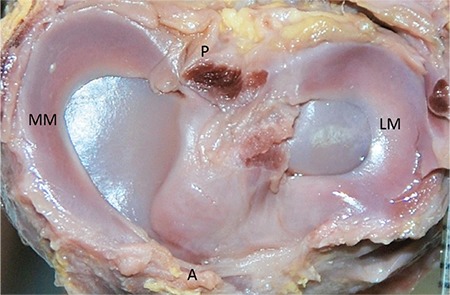
32-week-old foetus, the LM C shaped, the MM sickle shaped. LM: lateral meniscus; MM: medial menisci

**FIG. 5.**
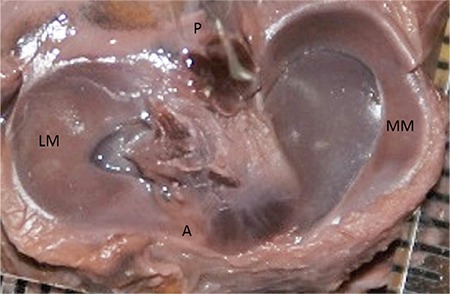
27-week-old foetus, the LM incomplete discoid, the MM sickle shaped. LM: lateral meniscus; MM: medial menisci

**FIG. 6.**
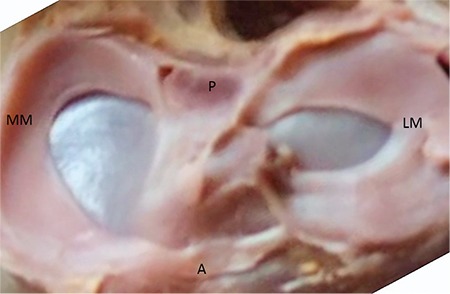
26-week-old foetus, the LM C shaped, the MM U shaped. LM: lateral meniscus; MM: medial menisci

**FIG. 7.**
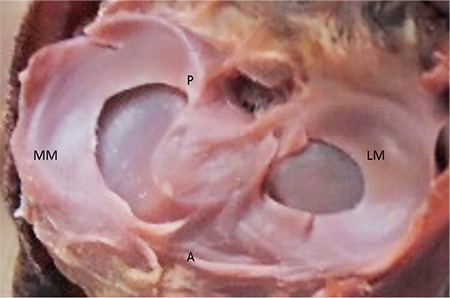
19-week-old foetus, the LM C shaped, the MM C shaped. LM: lateral meniscus; MM: medial menisci

**FIG. 8.**
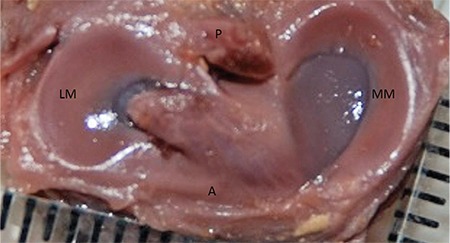
20-week-old foetus, the LM complete discoid, the MM crescentic shaped. LM: lateral meniscus; MM: medial menisci

Lateral and MM were classified morphologically (Figure 3,4,5,6,7,8). The most common shape of the MM was crescentic (50%), followed by sickle shaped (22.9%), V-shaped (10.9%), U-shaped (9.5%) and C-shaped (6.7%). The LM, on the other hand, was most commonly C-shaped (61%), followed by crescentic (19%), incomplete discoid (18.6%) and complete discoid (1.4%) (Table 4).

Male and female comparisons were carried out for all parameters, and there were no significant differences between the right- and the left-sided parameters.

## DISCUSSION

Menisci develop by differentiation of the mesenchymal tissue at the lower limb bud. They appear during the fourth week of human development, become evident at 9 weeks and assume the adult form at 14 weeks of gestation (7,10,13,14). The menisci are fibrous cartilage structures which reduce the contact stress sustained by the knee joint and contribute to congruence during stance and gait (1,2,3,4,5).

Studies employing different methods have been carried out on foetuses or adults to establish the type, area and location of the menisci. In a study carried out on 41 foetuses, Fukazawa et al. (4) measured the areas of the medial and lateral menisci and found that these areas increased with gestational age (4). The authors also reported that the area of the LM was greater than that of the MM, although the difference was not statistically significant (4). Tena-Arrequi et al. (13) also reported that the LM was larger than the MM during the foetal period (13). In a study in adults, Bloecker et al. (15) found a similar result, with the LM being larger than the MM. In the present study, both the lateral and medial meniscal areas were measured, and we found a statistically significant increase in the meniscal areas during the foetal period. Moreover, we found that the lateral was larger than the medial meniscal area, but the difference was not statistically significant. Our results were consistent with those of previous studies on foetuses and adults (4,13,15).

Fukazawa et al. (4) measured the medial and lateral articular surface areas of the tibia during the foetal period and found the medial articular surface areas to be larger than the lateral ones (4). The authors determined the ratios of the meniscal to the articular surface areas of the tibia to be 0.80 and 0.60 for the lateral and medial sides, respectively (4). They reported that because the medial articular surface area was larger than the lateral one, the LM occupied a relatively larger lateral articular surface area of the tibia (4). Larger medial vs. lateral articular surface areas of the tibia have also been documented in adults (15). In the present study, we found that the areas of the lateral and medial articular surfaces of the tibia were increased throughout the foetal period, and the medial articular surface areas of the tibia were larger than the lateral ones (p<0.05, Table 1). Moreover, the ratio of the lateral meniscal area to the lateral articular surface area was 0.78, whereas that of the medial meniscal area to the medial articular surface area was 0.61. Our finding that the LM occupied a larger area over the lateral articular surface during foetal life agreed with those of Fukazawa et al. (4).

We measured the distances between the anterior and posterior horns of the lateral and MM separately. The distance between the anterior and posterior horns of the MM was larger than that of the LM. In other words, the tips of the horns of the MM were farther apart. Therefore, we did not observe any V-shaped or U-shaped LM, nor did we observe complete or incomplete discoid type MM. We also measured the respective distances between the anterior and posterior horns of the menisci. The mean distance between the posterior horns was 2.66 mm, whereas the mean distance between the anterior horns was 4.71 mm, showing that the posterior horns are closer to each other than the anterior horns. A literature review did not reveal any studies in which the distance between the horns on both sides was measured. As such, this is the first study of its kind in the literature and would serve as a database for future studies.

The variations observed in menisci can be explained by their patterns of development in the embryologic and foetal periods (16,17). The medial and lateral condyles of the femur and tibia, which are involved in the knee joint, are not on the same anatomical plane (4). Femorotibial incongruence can be one of the reasons for many clinical problems, especially MM injuries. It has been argued that this asymmetric structure of the knee resulted in different shaped menisci (4). The shape of the LM is considerably different to that of the MM. Murlimanju et al. (3) classified the menisci and reported that the MM was most commonly crescentic (46.2%), whereas the LM was C-shaped (62.3%) during the foetal period (6). On the other hand, Kale et al. (8) also examined and classified the menisci according to their shapes and reported that the MM was most frequently sickle-shaped (36.36%), whereas the LM was incomplete discoid (54.54%). These are the only two published studies that classified the shapes of the menisci. In a study on adults, the MM was found to be crescentic in 50% of cases, whereas the LM was C-shaped in 61% of cases (18). In the present study, the most common shapes of the medial and lateral menisci were crescentic (50%) and C-shaped (61%), respectively (Table 5).

When all these studies were reviewed together, the MM and LM were found to have five and four different shapes, respectively (3,8,9) (Figure 2, Table 5). None of the three studies noted complete and incomplete discoid types among MM nor the U-shape, V-shape or sickle-shape among LM (3,8,9). Our findings and those of Murlimanju agree in that the MM was crescentic, and the LM was C-shaped. On the other hand, our findings contrasted with those of Kale et al. (8), who reported that the MM was most commonly sickle-shaped, whereas the LM was of the incomplete discoid type. This discrepancy can be attributed to the smaller sample size and the inclusion of only full-term foetuses in the study by Kale et al. (8).

Discoid meniscus was first defined in 1889, and it has been argued that it is observed only in the LM (4,6,8,9,19). The frequency of discoid meniscus in adults varies between 0 and 7% in cadaveric studies and between 0.4% and 16.6% in arthroscopic studies (7,8,16). Interracial differences in this frequency have been reported, with a slightly higher value observed in Asian populations (4,6,20). There are very few publications on the morphological development of discoid meniscus, and some authors consider discoid meniscus as a developmental anomaly (2,7,21). A deficiency in the resorption of the centre of the cartilage plate during foetal development and a genetic trait have been proposed as mechanisms responsible for the development of discoid meniscus (2,7,10,17,18,22,23,24).

Complete and incomplete discoid types have been reported only in the LM in previous studies (8,9). Our study confirmed this finding. The incomplete discoid type was observed in 18.6%, whereas the complete discoid type was found in 1.4% of all cases. This ratio is comparable to that found by Murlimanju et al. (9), whereas Kale et al. (8) reported a higher value for this ratio, which could have been due to the small number of foetuses in their study. In the present study, incomplete and complete discoid types were more common in the early foetal period. The complete discoid type was not observed in full-term foetuses, and the incomplete discoid type was observed in only one case. Based on our results, we can conclude that the frequency of complete and incomplete discoid types decreases with gestational age. Our study shows that meniscal differentiation can begin earlier during the intrauterine period. As previously mentioned, differences in the shape of the meniscus may be due to mesenchymal differentiation or to the development of the vasculature early in embryonic life.

In conclusion, we believe that the results of the present study enable us to fully understand the pathologies and anomalies of the menisci and contribute to diagnosis and treatment of these conditions as well as future scientific studies.

## Figures and Tables

**Table 1:**
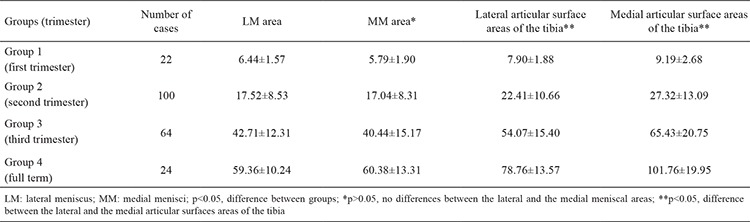
Mean ± standard deviations of the LM area, MM area and superior lateral and medial articular surface areas of the tibia in each group (mm^2^)

**Table 2:**
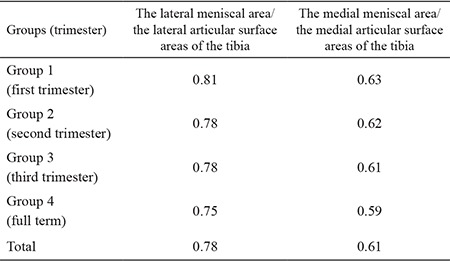
The ratio of the lateral and medial meniscal area to the lateral and medial articular surface areas of the tibia

**Table 3:**
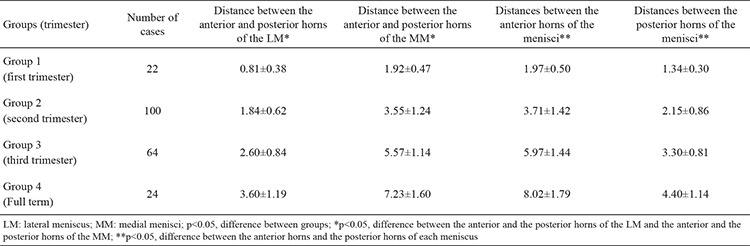
Means ± standard deviation of the distance between the anterior and posterior horns of the lateral and medial menisci, and between the anterior horns of the menisci and the posterior horns of the menisci (mm)

**Table 4:**
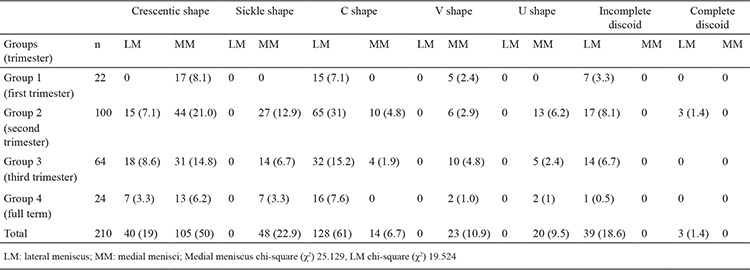
Number of cases and percentage distribution of the types of LM and MM in each group [n, (%)]

**Table 5:**
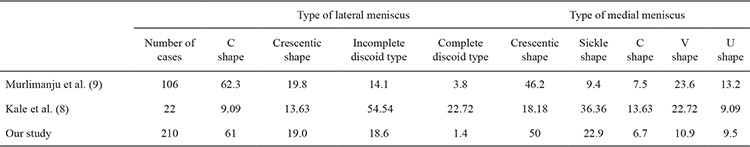
Comparison of the current results with those of previous studies (%)
